# Supplementary material for: Automated identification of diagnostic labelling errors in medicine
Source: Diagnosis (Berl). 2021 Oct 21;9(2):241–9. doi: 10.1515/dx-2021-0039 (PMC9125795; doi:10.1515/dx-2021-0039)
Supplement: Supplementary file 1 — Supplementary Material Details [file j_dx-2021-0039_suppl_001.docx]

**TITLE: Automated identification of diagnostic labelling errors in medicine**

**Running head: Automated identification of diagnostic labelling errors**

**AUTHORS:** Wolf E Hautz^1^, Moritz M Kündig^2^*, Roger Tschanz^2^*, Tanja Birrenbach^1^, Alexander Schuster^3^, Thomas Bürkle^2^, Stefanie C Hautz^1^, Thomas C Sauter^1#^, Gert Krummrey^1#^

^1^ Department of Emergency Medicine, Inselspital University Hospital, University of Bern, Bern, Switzerland
^2^ Berner Fachhochschule, Biel, Switzerland
^3^ Jung-Stilling-Klinikum, Siegen, Germany

*contributed equally, # contributed equally

**Corresponding author:**

Prof. Dr. med. Wolf Hautz MME
Department of Emergency Medicine
Inselspital University Hospital
Freiburgstrasse
3010 Bern
Switzerland
Phone: +41 31 632 57 01
Fax: +41 31 632 24 50
Email: [wolf.hautz@insel.ch](mailto:wolf.hautz@insel.ch)

**WORD COUNT:** 3531

**Tables:** 3 **Figures:** 2 **Supplemental material**: yes (two tables)

**ABSTRACT**

**Introduction** Identification of diagnostic error is complex and mostly relies on expert ratings, a severely limited procedure. We developed a system that allows to automatically identify diagnostic labelling error from diagnoses coded according to the international classification of diseases (ICD), often available as routine health care data.

**Methods** The system developed (index test) was validated against rater based classifications taken from three previous studies of diagnostic labeling error (reference standard). The system compares pairs of diagnoses through calculation of their distance within the ICD taxonomy. Calculation is based on four different algorithms. To assess the concordance between index test and reference standard, we calculated the area under the receiver operating characteristics curve (AUROC) and corresponding confidence intervals. Analysis were conducted overall and separately per algorithm and type of available dataset.

**Results** Diagnoses of 1127 cases were analyzed. Raters previously classified 24.58% of cases as diagnostic labelling errors (ranging from 12.3% to 87.2% in the three datasets). AUROC ranged between 0.821 and 0.837 overall, depending on the algorithm used to calculate the index test (95% CIs ranging from 0.8 to 0.86). Analyzed per type of dataset separately, the highest AUROC was 0.924 (95%CI 0.887-0.962).

**Conclusion** The trigger system to automatically identify diagnostic labeling error from routine health care data performs excellent, and is unaffected by the reference standards’ limitations. It is however only applicable to cases with pairs of diagnoses, of which one must be more accurate or otherwise superior than the other, reflecting a prevalent definition of a diagnostic labeling error.

**KEYWORDS:** diagnostic error, quality improvement, decision support

**INTRODUCTION**

Diagnostic error is a frequent health care problem (1–4) with major medical,(4–6) legal (7–9) and economic consequences.(10) On average, every patient in the U.S. experiences one major diagnostic error throughout his or her lifetime, often with devastating consequences for themselves, their families and their health care providers.(11) Thus, research into causes and predictors of diagnostic error as well as interventions to achieve diagnostic excellence is urgently needed.

In the existing research into the phenomenon, two conceptions of diagnostic error prevail.(12,13) One strand of research evaluates lapses in the diagnostic *processes* and often aims to identify missed opportunities.(14) Indeed, record reviews have found that in both, primary care and internal medicine, errors in the diagnostic process are common (15,16) and often not limited to just one process error per case. For example, Singh and colleagues, in a review of cases identified through an automated flagging of electronic health records (a technique called e-triggers (17)), found that 43.7% of erroneous cases in primary care involved more than one type of process breakdown.(15) Similarly, in a review of cases identified through autopsy discrepancies, quality assurance activities, and voluntary reports, Graber and colleagues identified 5.9 different process errors per case.(16) However, only some of these errors resulted in a wrong diagnostic label, and/or harm from delayed or wrong treatment. In his conceptual model of missed opportunities in diagnosis, Singh accounts for this phenomenon and distinguishes between four types of errors. (18) These are missed opportunities in diagnosis that did not result in patient harm, those that did (an entity he consequently terms “preventable diagnostic harm”), and delayed/wrong diagnosis without clear evidence of a missed opportunity, either with or without resulting harm. (18) This latter group of wrong diagnosis without clear evidence of a missed opportunity can be referred to as diagnostic *labelling* errors - the second major conception of diagnostic error (i.e. naming a disease A while in fact, it is B).(12) A labelling error does not necessarily imply existence of a causative process error, (12) but may instead also result from what Graber labelled “no-fault errors”, including masked or unusual disease presentations, or error due to uncooperative or deceptive patients.(16) Identification of a diagnostic labelling error should thus trigger a case review, because arguably, only those labelling errors that result from a process error are readily susceptible to interventions. However, the labelling error is what ultimately affects treatment and prognosis and thus results in suboptimal patient outcome.(13) Consequently, identification (and ultimately prevention) of labelling errors is of major importance for the patient. It is, however, only a first step on the way to identify addressable underlying causes. In that regard, identification of diagnostic labelling errors can be viewed as another trigger within Singh’s concept of them (other triggers being e.g. unscheduled revisits (17) or elevated PSA values without timely follow-up (19)). Each case identified by such a trigger should then lead to an in-depth review to identify and potentially rectify underlying process errors. Such a strategy would substantially lower the workload of chart review, because labelling errors have been identified in between 2 and 35 % of cases, depending on medical speciality and context.(20)

Existing research into labelling errors often compares first diagnoses to final, confirmed or otherwise more secure diagnoses. Next to triggering chart reviews, studies evaluating concordance between first and later diagnoses have also been used to identify associations between labelling errors and hospital length of stay,(4,21) mortality, (4,22) costs,(23) or type and size of hospital.(24) Such comparisons are typically conducted by a group of expert raters, a method with several disadvantages:

- The method is tedious, requires substantial expert contribution and thus does not scale well to larger datasets.
- Rater based identification of labelling errors are subject to hindsight-, and outcome bias.(25)
- Rater agreement is often limited.(4,25,26)
- The procedure introduces a substantial temporal delay between a patient being seen and the evaluation of his or her diagnosis, because it requires rater recruitment, training and the actual ratings.

Taken together, all the above disadvantages explain why measurement of diagnostic labelling errors is hardly incorporated into routine quality evaluations in healthcare and has not been established as another trigger tool in diagnostic safety management and research.

Beyond the field of labelling errors in clinical practice, rater based comparison of actual versus correct diagnoses is also used in competence assessments of health care professionals, research into such assessments and research into the cognitive psychology of diagnostic reasoning. Here, case vignettes are frequently used (e.g. (27–30)) and a known, correct diagnosis for each vignette is then compared to the actual diagnosis of study participants. Similar designs are used when evaluating the accuracy of diagnostic decision support systems (for a review see (31)) and patient employed symptom checkers (for a review, see (32)). All of the above research requires comparison of two diagnoses and their classification as either identical or discrepant. Thus, the shortcomings of rater based comparisons of two diagnoses are relevant to a large audience, and to fields of research as different as diagnostic quality management, health professions assessment and medical decision support.

To address the shortcomings of rater based identification of diagnostic labelling errors, we developed an automated scoring system. The system (i) compares pairs of diagnoses, (ii) quantifies their degree of discrepancy and (iii) allows classifying them as either similar or discrepant, based on pre-specified values for either sensitivity or specificity of such classifications. Here, we describe and validate this automated scoring system.

**METHODS**

**Classification of disease similarity**

We developed a web based computer program that compares pairs of diagnoses coded according to WHOs international classification of diseases (ICD). The similarity between two given ICD-codes is determined in four different ways, described in the following.

The current ICD version 10 is a uniaxial and non-hierarchical classification system, which contains 22 disease chapters (e.g. chapter IV: metabolic diseases), subdivided into 261 disease groups (e.g. E10 to 14: diabetes mellitus). These are further subdivided into 2’037 categories (e.g. E 10.-: primary insulin dependent diabetes mellitus), finally classifying each disease by one of 12’161 specific four-digit codes (e.g. E10.1: primary insulin dependent diabetes mellitus with ketoacidosis). The whole classification system can be depicted as a graph (figure 1). A simple way (termed *steps* in the following) to determine the similarity of two given diseases is to count the edges on the shortest path connecting them (depicted as a dashed line in figure 1).
A slightly more complex approach allows adjusting for medical similarity by assigning different weights for each type of edge (for an example, see figure 1). For example, one could argue that misdiagnosing one metabolic disease as another metabolic disease is less problematic then classifying a metabolic disease as a neoplasm. This medical similarity can be represented by assigning the edges connecting chapters of the ICD a higher weight than the edges connecting diseases within a chapter (depicted by the bold line in figure 1). Similarity of two diseases is then determined by summing the weights of the edges on the shortest path connecting the two diseases (an approach termed *weights* below).

Two more approaches originate from research into natural language processing. The approach devised by Wu and Palmer (thus labelled *WuPalmer* below) accounts for the position of two given concepts in the classification hierarchy relative to the position of their least common subsumer (i.e. their most specific common ancestor).(33,34) The score devised by Li and colleagues (termed *Lietal*) adapts this approach by combining the shortest path length and the depth of the subsumer nonlinearly, thus better reflecting human similarity ratings.(35,36)

**Validation**

To assess the performance of each of the four different algorithms *steps*, *weights*, *WuPalmer* and *Lietal*, we applied each one to three different datasets, two of which have been previously published. Each of these three datasets contains pairs of diagnoses that were previously rated by at least two experts as either similar or discrepant, a rating we used as reference standard to evaluate the four algorithms against. Notably, it is the free text diagnoses that were assessed by raters as similar or discrepant, not their ICD codes.
The first dataset, termed *clinical*, contains first and final primary diagnoses of 755 patients that presented to an emergency room and were subsequently hospitalized to an internal medicine ward.(4) Patients presented with a broad variety of specific and non-specific chief complaints (26) and were diagnosed with a multitude of conditions across the spectrum of internal medicine. Three trained raters independently classified the pair of ER admittance diagnosis and internal medicine discharge diagnosis as either similar or discrepant, based on a previously validated scheme.(37) Discrepancies between raters were subsequently discussed and resolved.

The second and third dataset resulted from studies of clinical reasoning in advanced medical students and are thus termed *educational*. The second dataset stems from a study of 20 advanced medical students who each diagnosed six virtual patients with shortage of breath for a variety of reasons in a previously validated computerized assessment of diagnostic reasoning,(38) resulting in 120 pairs of diagnoses. The true diagnosis of each of these six patients has been validated by 20 expert physicians.(38) Three expert raters scored each pair of diagnoses as either similar or discrepant, disagreements were again resolved by discussion and consensus.
The third dataset stems from a study of 51 advanced medical students who each diagnosed 8 virtual patients either individually (17 students) or in pairs of two (34 students in 17 pairs), resulting in a total of 34 * 8 = 272 pairs of diagnoses. Chief complaints and correct diagnoses were dispersed across the spectrum of internal medicine. Again, three expert raters scored each pair of diagnoses as either similar or discrepant, disagreements were resolved by discussion and consensus.

All diagnoses used in the study were ICD coded by one of two trained physicians. After coding a random sample of 10% of all diagnoses independent and in duplicate according to a predefined scheme, we assessed their coding agreement as excellent (kappa = 0.96). The remaining diagnoses were then evenly split between raters and coded individually.

Because this paper reports a reanalysis of previously collected datasets, we did not obtain ethical approval for the conduct of this study. The three original studies collecting the datasets used here have all obtained ethical approval and informed participant consent.

**Statistical analysis**

The existing rater based evaluations of pairs of diagnoses as either discrepant or similar were used as reference standard. We quantified semantic similarity of the diagnoses within each pair through each of the four approaches detailed above (*steps*, *weights*, *WuPalmer and Lietal*) and computed sensitivity and specificity for each of these approaches in comparison to the reference standard. We calculated receiver operating characteristics (ROC) curves, and estimated the area under the curve (AUC) and its 95 % confidence interval. We further tested for a significant difference towards the null hypothesis (i.e. that the approach under evaluation is no better than throwing a coin in determining whether two given diagnoses are discrepant). All analyse were computed in IBM SPSS Statistics version 25 and conducted for the *clinical* end *educational* datasets separately as well as for the pooled data from all of the validation datasets.

**RESULTS**

The web based computer program that compares pairs of diagnoses coded according to the ICD is available for free and public use online at [https://simple.right-icd.de](https://right-icd.de). The program supports diagnoses coded in different versions of the ICD, such as versions 9 and 10. The following results were obtained for diagnoses coded in ICD, German modification, version 10.(39), which is similar to other versions of ICD 10 (such as e.g. the “clinical modification” frequently employed in the U.S.) with regard to the four digit codes of diagnoses.

A total of 1172 pairs of diagnoses were available for analysis (table 1). In the second dataset, 11 students only provided a diagnosis in 5 out of 6 cases, resulting in 109 out of 120 pairs of diagnoses being available for analysis.

[Please insert table 1 here]

The performance (i.e. the ability to discriminate between similar and discrepant diagnoses) of all four algorithms evaluated was high, ranging from an AUC of 0.821 to 0.835 (table 2). The corresponding ROC curves are depicted in figure 2. AUC further increases when educational and clinical datasets are analysed separately. The highest AUC observed was 0.924 for the weights algorithm applied to the educational datasets. No AUC was below 0.82 (table 2).

[Please insert table 2 here.]

All four investigated algorithms calculate continuous values, such as the number of steps required within the ICD taxonomy to get from one diagnosis to another. Conversion of these numerical values into a dichotomous decision (i.e. discrepant or similar) requires a cut-off value, below which two diagnoses are classified as similar and above which they are classified as discrepant. (Note that this statement is only true for the algorithms steps and weights, where smaller numbers indicate higher similarity. For WuPalmer and Lietal, larger numbers indicate higher similarity. Consequently, for the latter two algorithms, the cut off represents the value *below* which two diagnoses are classified as discrepant.) Table 3 allows to choose appropriate cut-off values based on a choice of sensitivity (or specificity). Tables S1 and S2 in the supplemental information provide similar cut off values for clinical and educational datasets separately.

[Please insert table 3 here.]

**DISCUSSION**

“*Improving the diagnostic process […] represents a moral, professional, and public health imperative.”*(11) At present, however, “*our health care systems are unable to systemically measure diagnostic performance […], which limits the ability to quantify performance and guide improvements”*.(40) Furthermore, because diagnostic error is rarely measured outside of research studies, we may underestimate the magnitude of the problem. In effect, health care organizations do not have insights into the diagnostic quality they provide, and quality improvement initiatives lack measures of their efficacy and efficiency. The approach presented here represents a step towards routine measurement of diagnostic labelling errors at a systemic level and scale.

In this study, we present and validate an approach that allows to automatically identify diagnostic labelling errors from routinely available healthcare data, namely ICD coded diagnoses. The approach performs quite well when compared to human classifications of diagnostic labelling errors, resulting in an AUC ranging from 0.821 to 0.924. Its performance is largely independent of the prevalence of labelling error in the data evaluated, which ranged from 12.3% in the clinical to 87.2% in one of the educational the datasets used for validation. Furthermore, it can be applied to datasets of any size, and the sensitivity (or specificity) for the identification of diagnostic labelling errors can be tailored to the specific task at hand. Manual identification of diagnostic labelling errors would only allow to adjust sensitivity or specificity to a given purpose, if raters graded the amount of discrepancy, e.g. using Likert scales, which would further add to their workload. Thus, most studies of diagnostic labelling errors employ dichotomous ratings instead (see e.g. (4,21–23)). However, adjusting sensitivity (or specificity) to the studies purpose may be beneficial. For example, when used as a trigger in clinical quality assurance or research into diagnostic error in a clinical setting, one would most likely prioritize sensitivity over specificity, in order to identify all cases with potential for improvements. In summative assessments however (i.e. assessments resulting in pass or fail decisions), one could argue that coding of diagnostic labelling errors should prioritize specificity over sensitivity to support defensible fail decisions.

Because of the wide spread use of Grabers’ definition of a diagnostic error as *“a diagnosis that was unintentionally delayed […], wrong […], or missed […], as judged from the eventual appreciation of more definitive information”,*(16) diagnostic errors are conceptualized as a discrepancy between a first and a more definitive diagnosis across a wide range of research and applications. This makes our approach applicable to many fields, from routine quality assurance to decision-making research, and from the evaluation of decision support systems to the scoring of assessments.

Our approach complements existing methods used in research into diagnostic error. The limitations of rater based assessments of diagnostic error, including hindsight-, and outcome bias,(25) limited rater agreement (4,25,26) and their limitation to small sample sizes have been pointed out in the introduction. None of these shortcomings applies to the approach suggested here. It should however be noted that simply only identifying a discrepancy between two diagnostic labels is insufficient to identify an underlying diagnostic process error. Instead, a discrepancy between diagnostic labels should be interpreted as a trigger to review the case in more detail to identify potentially preventable process errors and because a discrepancy between a first and a later diagnostic label may also result from disease evolution, or development of a secondary complication. Such discrepancies may not represent an error (not even a labelling error) at all. To account for these problems, combining our automated identification of diagnostic labelling errors with approaches such as the Symptom-Disease Pair Analysis of Diagnostic Error (SPADE) may improve the specificity of our trigger: in brief, SPADE looks out for “well-known diagnostic error dyads” such as dizziness – stroke or back pain – aortic aneurysm.(41) Limiting comparison of diagnostic labels with our approach to those labels that share a common symptom or chief complaint may potentially flag more diagnostic discrepancies as a labelling error where an underlying process error can be identified.

In general, electronic trigger tools watch out for predefined signals in electronic health records, indicative of diagnostic error or of suboptimal diagnostic performance.(42) Triggers can be generic (such as unscheduled revisits or visits followed by an unplanned hospitalization within a specified time frame (17)) or disease specific (such as screening for abnormal prostate-specific antigen (PSA) measurements without a timely follow-up action (19)). Such trigger tools can be applied to very large datasets and substantially reduce the number of cases to then manually check for diagnostic process error (e.g. from 292’587 records screened to 426 abnormal PSA measurements without follow-up (19)). However, manual work-up of the cases identified is still required. While the extent to which these triggers are associated with missed diagnostic opportunities (or process errors) has been established for the two aforementioned triggers, (17) similar work on the predictive value of labelling discrepancies remains to be done. Nevertheless, diagnostic labelling errors have been associated with adverse outcomes such as increased mortality or length of hospital stay before,(4,21,22,43) likely because it ultimately is the diagnostic label assigned to a patient that determines (in-)adequate therapy and prognosis.(13)

Researchers and quality managers who consider to use the approach and/or program presented here can easily validate it for their specific purpose, provided they do possess a dataset for validation, i.e. containing a reference classification against which to evaluate the approach and program. By running their datasets of pairs of ICD coded diagnoses through the web based program provided at <https://simple.right-icd.de>, they can not only calculate the AUC the different algorithms achieve for their validation datasets, but can also determine cutoff values to achieve a given sensitivity (or specificity) for their specific classification task. Publication of their findings can help to empirically determine the use cases for which the suggested approach and program is suitable and where reasonable borders of its applicability are. In addition, such revalidation attempts may help to identify differences between the four different algorithms evaluated here and implemented on the project website. In the three datasets used in the current study, all four algorithms performed remarkably similar. We do not see any operational advantage of one over the other, but would recommend to use the steps algorithm as a default, simply because it is the least complicated and most comprehensible approach.

**Limitations**

The approach presented in this paper and its validation have several limitations, which warrant consideration.

First, the approach is limited to datasets of pairs of diagnoses, of which one should be more certain, of better quality or otherwise superior than the other is.
Second, currently the program is limited to comparing pairs of single diagnoses. In theory, the approach can be applied to the comparison of two sets of one to many diagnoses each, and we are currently developing the program to account for that scenario. However, definition of a reference standard and thus validation become much more complex (and much less certain) in that scenario.
Third, the program is currently limited to the comparison of pairs of diagnoses coded in any of the more recent versions of ICD, making it susceptible to all the limitations that are inherent to ICD coding, and requiring ICD coding of all diagnoses to start with.
Last, we only validated our approach and program on medical cases. Weather our approach also works in e.g. neurological or paediatric cases remains to be tested. As pointed out above, researchers in possession of an ICD coded set of pair of diagnoses from any field can easily check the performance of our approach online.

**CONCLUSION**

In comparison to the reference standard, the trigger system developed to automatically identify diagnostic labelling error from routine health care data performs excellent, and is unaffected by the reference standards’ limitations. It is however only applicable to cases with pairs of diagnoses, of which one must be better, more accurate or otherwise superior than the other, reflecting the prevalent definition of a diagnostic labeling errors in medicine.

**REQUIRED STATEMENTS**

**Data availability**

The datasets analysed for validation of the proposed algorithm stem from previously published studies and are available from the authors of the original publications.

**Acknowledgments** We would like to thank the editor of this paper, Dr. Hardeep Singh, and two anonymous reviewers for their constructive comments on a previous version of this paper.

**Competing Interests** WEH has received research funding from Mundipharma Research UK, Roche Diagnostics Germany, Dräger Medical Germany, MDI Australia and consultancy fees from AO foundation Zurich, all outside the work submitted.

**Funding** Funded by the Swiss National Science Foundation under grant number 187284, *The digital diagnostician: how information technology affects medical diagnoses.*

**REFERENCES**

1. Balogh EP, Miller BT, Ball JR, editors. Improving Diagnosis in Health Care [Internet]. Washington, D.C.: National Academies Press; 2015 [cited 2017 Apr 28]. Available from: http://www.nap.edu/catalog/21794

2. Leape LL, Brennan TA, Laird N, Lawthers AG, Localio AR, Barnes BA, et al. The nature of adverse events in hospitalized patients. Results of the Harvard Medical Practice Study II. N Engl J Med. 1991 Feb 7;324(6):377–84.

3. Thomas EJ, Studdert DM, Burstin HR, Orav EJ, Zeena T, Williams EJ, et al. Incidence and types of adverse events and negligent care in Utah and Colorado. Med Care. 2000 Mar;38(3):261–71.

4. Hautz WE, Kämmer JE, Hautz SC, Sauter TC, Zwaan L, Exadaktylos AK, et al. Diagnostic error increases mortality and length of hospital stay in patients presenting through the emergency room. Scand J Trauma Resusc Emerg Med. 2019 May 8;27(1):54.

5. Graber M. Diagnostic errors in medicine: a case of neglect. Jt Comm J Qual Patient Saf. 2005 Feb;31(2):106–13.

6. Newman-Toker DE. Diagnostic Errors—The Next Frontier for Patient Safety. JAMA. 2009 Mar 11;301(10):1060.

7. Brown TW, McCarthy ML, Kelen GD, Levy F. An epidemiologic study of closed emergency department malpractice claims in a national database of physician malpractice insurers. Acad Emerg Med. 2010 May;17(5):553–60.

8. Schaffer AC, Jena AB, Seabury SA, Singh H, Chalasani V, Kachalia A. Rates and Characteristics of Paid Malpractice Claims Among US Physicians by Specialty, 1992-2014. JAMA Internal Medicine. 2017 May 1;177(5):710.

9. Kachalia A, Gandhi TK, Puopolo AL, Yoon C, Thomas EJ, Griffey R, et al. Missed and delayed diagnoses in the emergency department: a study of closed malpractice claims from 4 liability insurers. Ann Emerg Med. 2007 Feb;49(2):196–205.

10. Weeks WB, Foster T, Wallace AE, Stalhandske E. Tort Claims Analysis in the Veterans Health Administration for Quality Improvement. The Journal of Law, Medicine & Ethics. 2001 Sep;29(3–4):335–45.

11. Committee on Diagnostic Error in Health Care, Board on Health Care Services, Institute of Medicine, The National Academies of Sciences, Engineering, and Medicine. Improving Diagnosis in Health Care [Internet]. Balogh EP, Miller BT, Ball JR, editors. Washington, D.C.: National Academies Press; 2015 [cited 2017 Apr 28]. Available from: http://www.nap.edu/catalog/21794

12. Newman-Toker DE. A unified conceptual model for diagnostic errors: underdiagnosis, overdiagnosis, and misdiagnosis. Diagnosis (Berl). 2014 Jan;1(1):43–8.

13. Hautz WE. When I say… diagnostic error. Medical Education. 2018 Jun 4;52:896–7.

14. Lyratzopoulos G, Vedsted P, Singh H. Understanding missed opportunities for more timely diagnosis of cancer in symptomatic patients after presentation. Br J Cancer. 2015 Mar;112(S1):S84–91.

15. Singh H, Giardina TD, Meyer AND, Forjuoh SN, Reis MD, Thomas EJ. Types and origins of diagnostic errors in primary care settings. JAMA Intern Med. 2013 Mar 25;173(6):418–25.

16. Graber ML, Franklin N, Gordon R. Diagnostic Error in Internal Medicine. Archives of Internal Medicine. 2005 Jul 11;165(13):1493.

17. Singh H, Giardina TD, Forjuoh SN, Reis MD, Kosmach S, Khan MM, et al. Electronic health record-based surveillance of diagnostic errors in primary care. BMJ Qual Saf. 2012 Feb;21(2):93–100.

18. Singh H. Editorial: Helping Health Care Organizations to Define Diagnostic Errors as Missed Opportunities in Diagnosis. The Joint Commission Journal on Quality and Patient Safety. 2014 Mar;40(3):99-AP1.

19. Murphy DR, Laxmisan A, Reis BA, Thomas EJ, Esquivel A, Forjuoh SN, et al. Electronic health record-based triggers to detect potential delays in cancer diagnosis. BMJ Qual Saf. 2014 Jan;23(1):8–16.

20. Berner ES, Graber ML. Overconfidence as a cause of diagnostic error in medicine. Am J Med. 2008 May;121(5 Suppl):S2-23.

21. Johnson T, McNutt R, Odwazny R, Patel D, Baker S. Discrepancy between admission and discharge diagnoses as a predictor of hospital length of stay. J Hosp Med. 2009 Apr;4(4):234–9.

22. Eames J, Eisenman A, Schuster RJ. Disagreement between emergency department admission diagnosis and hospital discharge diagnosis: mortality and morbidity. Diagnosis. 2016 Mar 1;3(1):23–30.

23. McNutt R, Johnson T, Kane J, Ackerman M, Odwazny R, Bardhan J. Cost and Quality Implications of Discrepancies Between Admitting and Discharge Diagnoses. Quality Management in Health Care. 2012 Oct;21(4):220–7.

24. Battle RM, Pathak D, Humble CG, Key CR, Vanatta PR, Hill RB, et al. Factors influencing discrepancies between premortem and postmortem diagnoses. JAMA. 1987 Jul 17;258(3):339–44.

25. Zwaan L, Monteiro S, Sherbino J, Ilgen J, Howey B, Norman G. Is bias in the eye of the beholder? A vignette study to assess recognition of cognitive biases in clinical case workups. BMJ Qual Saf. 2017;26(2):104–10.

26. Sauter TC, Capaldo G, Hoffmann M, Birrenbach T, Hautz SC, Kämmer JE, et al. Non-specific complaints at emergency department presentation result in unclear diagnoses and lengthened hospitalization: a prospective observational study. Scand J Trauma Resusc Emerg Med. 2018 Dec;26(1):60.

27. Kämmer JE, Hautz WE, März M. Self-monitoring accuracy does not increase throughout undergraduate medical education. Med Educ. 2020;54(4):320–7.

28. Mamede S, Hautz WE, Berendonk C, Hautz SC, Sauter TC, Rotgans JI, et al. Think twice: Effects of Returning to the Case to Reflect upon the Initial Diagnosis on Diagnostic Accuracy. Acad Med. 2019;in press.

29. Hautz WE, Schubert S, Schauber SK, Kunina‐Habenicht O, Hautz SC, Kämmer JE, et al. Accuracy of self‐monitoring: does experience, ability or case difficulty matter? Med Educ. 2019 Jul;53(7):735–44.

30. Hautz WE, Kammer JE, Schauber SK, Spies CD, Gaissmaier W. Diagnostic performance by medical students working individually or in teams. JAMA. 2015 Jan 20;313(3):303–4.

31. Riches N, Panagioti M, Alam R, Cheraghi-Sohi S, Campbell S, Esmail A, et al. The Effectiveness of Electronic Differential Diagnoses (DDX) Generators: A Systematic Review and Meta-Analysis. Schmidt RL, editor. PLOS ONE. 2016 Mar 8;11(3):e0148991.

32. Fraser H, Coiera E, Wong D. Safety of patient-facing digital symptom checkers. The Lancet. 2018 Nov;392(10161):2263–4.

33. Wu Z, Palmer M. Verbs semantics and lexical selection. In: Proceedings of the 32nd annual meeting on Association for Computational Linguistics - [Internet]. Las Cruces, New Mexico: Association for Computational Linguistics; 1994 [cited 2021 Jan 3]. p. 133–8. Available from: http://portal.acm.org/citation.cfm?doid=981732.981751

34. Jia Z, Lu X, Duan H, Li H. Using the distance between sets of hierarchical taxonomic clinical concepts to measure patient similarity. BMC Med Inform Decis Mak. 2019 Dec;19(1):91.

35. Yuhua Li, Bandar ZA, McLean D. An approach for measuring semantic similarity between words using multiple information sources. IEEE Transactions on Knowledge and Data Engineering. 2003 Jul;15(4):871–82.

36. Girardi D, Wartner S, Halmerbauer G, Ehrenmüller M, Kosorus H, Dreiseitl S. Using concept hierarchies to improve calculation of patient similarity. Journal of Biomedical Informatics. 2016 Oct;63:66–73.

37. Hautz SC, Schuler L, Kämmer JE, Schauber SK, Ricklin ME, Sauter TC, et al. Factors predicting a change in diagnosis in patients hospitalised through the emergency room: a prospective observational study. BMJ open. 2016;6(5):e011585.

38. Kunina-Habenicht O, Hautz WE, Knigge M, Spies C, Ahlers O. Assessing clinical reasoning (ASCLIRE): Instrument development and validation. Advances in Health Sciences Education. 2015 Dec;20(5):1205–24.

39. Deutsches institut für Medizinische Dokuentation und Information. Internationale statistische Klassifikation der Krankheiten und verwandter Gesundheitsprobleme, 10. Revision, German Modification, Version 2020 [Internet]. ICD-10-GM Version 2020. 2020 [cited 2020 Dec 11]. Available from: https://www.dimdi.de/static/de/klassifikationen/icd/icd-10-gm/kode-suche/htmlgm2020/

40. Gordon and Betty Moore Foundation. Improving diagnostic performance to reduce harm, improve health outcomes and save lives. [Internet]. [cited 2020 Dec 11]. Available from: https://www.moore.org/initiative-strategy-detail?initiativeId=diagnostic-excellence

41. Liberman AL, Newman-Toker DE. Symptom-Disease Pair Analysis of Diagnostic Error (SPADE): a conceptual framework and methodological approach for unearthing misdiagnosis-related harms using big data. BMJ Qual Saf. 2018 Jul;27(7):557–66.

42. Murphy DR, Meyer AN, Sittig DF, Meeks DW, Thomas EJ, Singh H. Application of electronic trigger tools to identify targets for improving diagnostic safety. BMJ Quality & Safety. 2018 Oct 5;bmjqs-2018-008086.

43. Bastakoti M, Muhailan M, Nassar A, Sallam T, Desale S, Fouda R, et al. Discrepancy between emergency department admission diagnosis and hospital discharge diagnosis and its impact on length of stay, up-triage to the intensive care unit, and mortality. Diagnosis. 2021 Jul 5;0(0):000010151520210001.

| **Data-set** | **type** | **Size** | **pairs of diagnoses*** | **Cases with diagnoses** | | **Prevalence of error** |
| --- | --- | --- | --- | --- | --- | --- |
|  |  |  |  | **similar** | **discrepant** |  |
| 1 | clinical | 755 patients, admission and discharge diagnosis | 755 (100%) | 662 | 93 | 12.30% |
| 2 | educational | 20 advanced students diagnosing 6 virtual patients each | 109 (90.34%) | 14 | 95 | 87.20% |
| 3 | educational | 51 advanced students diagnosing 8 virtual patients alone (17) or in pairs (34; 17 teams) | 272 (100%) | 180 | 92 | 66.2% |
| **total** | | | **1127 (98.26%)** | **850** | **277** | **24.58%** |

**Table 1: Datasets used to validate the automated identification of diagnostic labelling errors.** *percentages refer to pairs of diagnoses with complete data available for analysis.

|  | **Area under the curve AUC and (95% Confidence interval)** | | |
| --- | --- | --- | --- |
| **Algorithm** | **Overall** | **clinical dataset only** | **educational datasets only** |
| steps | 0.835 (0.812 - 0.859) | 0.853 (0.822 - 0.884) | 0.92 (0.88 - 0.959) |
| weights | 0.830 (0.806 - 0.854) | 0.856 (0.826 - 0.887) | 0.924 (0.887 - 0.962) |
| Lietal | 0.821 (0.797 - 0.845) | 0.856 (0.825 - 0.887) | 0.923 (0.855 - 0.962) |
| WuPalmer | 0.821 (0.797 - 0.846) | 0.856 (0.825 - 0.887) | 0.923 (0.855 - 0.962) |

**Table 2: Performance of four algorithms overall, and by type of dataset**

| **Sensitivity** | **Specificity** | **steps** | **weights** | **WuPalmer** | **Lietal** |  |
| --- | --- | --- | --- | --- | --- | --- |
|  |  | classify as discrepant if larger or equal to | | classify as discrepant if smaller or equal to | |  |
| 0.00 | 1.00 | 11.00 | 26.00 | -1.00 | -1.00 |  |
| 0.01 | 1.00 | 9.50 | 24.85 |  |  |  |
| 0.08 | 0.97 | 8.50 | 24.55 |  |  |  |
| 0.38 | 0.88 | 7.50 | 23.65 |  |  |  |
| 0.51 | 0.80 |  | 12.85 | 0.1 |  |  |
| 0.51 | 0.81 |  | 17.80 |  | 0.05 |  |
| 0.66 | 0.80 | 6.50 |  |  |  |  |
| 0.67 | 0.80 |  | 12.55 | 0.24 | 0.15 |  |
| 0.80 | 0.78 | 5.50 | 11.65 | 0.27 | 0.18 |  |
| 0.82 | 0.76 |  | 11.4 | 0.31 | 0.22 |  |
| 0.83 | 0.76 | 4.50 | 5.85 | 0.42 | 0.28 |  |
| 0.89 | 0.73 | 3.50 | 4.65 | 0.54 | 0.44 |  |
| 0.91 | 0.71 | 2.50 | 1.95 | 0.71 | 0.60 |  |
| 0.97 | 0.67 | 0.50 | 0.45 | 0.93 | 0.89 |  |
| 1.00 | 0.00 | -1.00 | -1.00 | 2.00 | 2.00 |  |

**Table 3: Cut-off values for all for algorithms to achieve a given sensitivity (or specificity).**

**FIGURE LEDGENDS**

**Figure 1: Example of an ICD taxonomy.** Dotted line: Distance between two given exemplary diagnosis within this taxonomy is determined by counting the edges of the shortest path connecting them. Bold line: alternatively, the edges on the shortest connecting path between any two diagnoses can be determined by summing the weights of the edges connecting them.

**Figure 2: Sensitivity and specificity to identify diagnostic labelling errors**, by algorithm and type of dataset
