# Supplementary material for: Automated identification of diagnostic labelling errors in medicine
Source: Diagnosis (Berl). 2021 Oct 21;9(2):241–9. doi: 10.1515/dx-2021-0039 (PMC9125795; doi:10.1515/dx-2021-0039)
Supplement: Supplementary file 2 — Supplementary Material Details [file j_dx-2021-0039_suppl_002.pdf]

Cut-off values				
Study type	Algorithm	classify as discrepant if larger or equal to	Sensitivity	1-Specificity
clinical	steps	-1.00	1.000	1.000
		0.50	0.978	0.358
		1.50	0.978	0.349
		2.50	0.968	0.316
		3.50	0.946	0.304
		4.50	0.903	0.266
		5.50	0.871	0.246
		6.50	0.785	0.218
		7.50	0.538	0.131
		8.50	0.086	0.032
		9.50	0.000	0.002
		11.00	0.000	0.000
	weights	-1.000	1.000	1.000
		0.450	0.978	0.358
		1.050	0.978	0.349
		1.500	0.978	0.347
		2.100	0.978	0.320
		3.000	0.978	0.316
		4.050	0.968	0.311
		4.950	0.957	0.305
		5.550	0.946	0.278
		5.850	0.946	0.276
		8.300	0.946	0.275
		10.950	0.935	0.270
		11.400	0.925	0.264
		11.650	0.892	0.246
		12.000	0.882	0.245
		12.300	0.860	0.243
		12.550	0.817	0.227
		12.850	0.817	0.218
		17.800	0.817	0.216
		23.050	0.785	0.207
		23.650	0.538	0.130
		24.100	0.495	0.124
		24.550	0.086	0.032
		24.850	0.000	0.002
		26.000	0.000	0.000
educational	steps	-1.00	1.000	1.000
		1.00	0.967	0.179
		2.50	0.887	0.160
		3.50	0.869	0.094
		4.50	0.800	0.066
		5.50	0.764	0.066
		6.50	0.611	0.066
		7.50	0.320	0.066
		8.50	0.084	0.000
		9.50	0.015	0.000
		11.00	0.000	0.000
	weights	-1.000	1.000	1.000
		0.900	0.967	0.179
		1.950	0.887	0.160
		2.250	0.884	0.104
		3.450	0.884	0.075
		4.650	0.869	0.066
		5.100	0.862	0.066
		5.550	0.804	0.066
		8.150	0.789	0.066
		11.050	0.785	0.066
		11.650	0.764	0.066
		12.100	0.760	0.066
		12.550	0.611	0.066
		12.850	0.404	0.066
		18.250	0.404	0.057
		23.650	0.320	0.057
		24.100	0.309	0.047
		24.550	0.084	0.000
		24.850	0.015	0.000
		26.000	0.000	0.000
