# Supplementary material for: Automated identification of diagnostic labelling errors in medicine
Source: Diagnosis (Berl). 2021 Oct 21;9(2):241–9. doi: 10.1515/dx-2021-0039 (PMC9125795; doi:10.1515/dx-2021-0039)
Supplement: Supplementary file 3 — Supplementary Material Details [file j_dx-2021-0039_suppl_003.pdf]

Cut-off values				
Study type	Algorithm	classify as discrepant if smaller or equal to	Sensitivity	1-Specificity
clinical	Wu & Palmer	-1.000	0.000	0.000
		0.100	0.849	0.224
		0.211	0.849	0.225
		0.236	0.849	0.234
		0.268	0.903	0.252
		0.310	0.935	0.270
		0.367	0.946	0.275
		0.422	0.946	0.276
		0.472	0.946	0.278
		0.536	0.957	0.305
		0.586	0.968	0.311
		0.633	0.968	0.316
		0.708	0.978	0.320
		0.804	0.978	0.349
		0.929	0.978	0.358
		2.000	1.000	1.000
	Li et al.	-1.000	0.000	0.000
		0.054	0.849	0.224
		0.120	0.849	0.225
		0.147	0.849	0.234
		0.180	0.903	0.252
		0.219	0.935	0.270
		0.246	0.946	0.275
		0.279	0.946	0.276
		0.341	0.946	0.278
		0.400	0.957	0.305
		0.441	0.957	0.310
		0.508	0.968	0.316
		0.597	0.978	0.320
		0.705	0.978	0.349
		0.888	0.978	0.358
		2.000	1.000	1.000
educational	Wu & Palmer	-1.000	0.000	0.000
		0.100	0.404	0.057
		0.211	0.404	0.066
		0.236	0.611	0.066
		0.268	0.764	0.066
		0.310	0.785	0.066
		0.389	0.789	0.066
		0.472	0.804	0.066
		0.536	0.869	0.066
		0.586	0.884	0.075
		0.633	0.884	0.104
		0.708	0.887	0.160
		0.875	0.967	0.179
		2.000	1.000	1.000
	Li et al.	-1.000	0.000	0.000
		0.054	0.404	0.057
		0.120	0.404	0.066
		0.147	0.611	0.066
		0.180	0.764	0.066
		0.219	0.785	0.066
		0.274	0.789	0.066
		0.341	0.804	0.066
		0.400	0.869	0.066
		0.441	0.869	0.094
		0.489	0.884	0.104
		0.577	0.887	0.160
		0.817	0.967	0.179
		2.000	1.000	1.000
